# Joint association of physical activity and diet quality with dyslipidemia: a cross-sectional study in Western China

**DOI:** 10.1186/s12944-024-02030-2

**Published:** 2024-02-10

**Authors:** Munire Mutalifu, Qian Zhao, Ying Wang, Xieyire Hamulati, Yu-Shan Wang, Lei Deng, Niyaziaili Adili, Fen Liu, Yi-Ning Yang, Xiao-Mei Li

**Affiliations:** 1 Department of Cardiology, State Key Laboratory of Pathogenesis, Prevention and Treatment of High Incidence Diseases in Central Asia, First Affiliated Hospital of Xinjiang Medical University, Urumqi, China; 2 Department of Cardiology, People’s Hospital of Xinjiang Uygur Autonomous Region, Urumqi, China; 3 Center of Health Management, The First Affiliated Hospital of Xinjiang Medical University, Urumqi, China; 4 Baoshihua Korla Hospital, Korla, China; 5 Xinjiang Key Laboratory of Cardiovascular Disease Research, Clinical Medical Research Institute of Xinjiang Medical University, Urumqi, China

**Keywords:** Dyslipidemia, Prevalence, Diet quality, Physical activity, Joint association

## Abstract

**Objective:**

This study aims to investigate the prevalence of dyslipidemia and assess the joint association of physical activity (PA) and diet quality on dyslipidemia risk in urban areas of Xinjiang.

**Methods:**

Conducted from July 2019 to September 2021 in Xinjiang, China, this cross-sectional study involved 11,855 participants (mean age 47.1 ± 9.4 years, 53.1% male). Standard methods were used to measure plasma cholesterol levels, and validated questionnaires were employed to evaluate dietary habits and PA. The definition of dyslipidemia is based on 2023 Chinese guidelines for lipid management. PA was divided into guideline-recommended moderate-to-vigorous physical activity (MVPA) and non-MVPA, following World Health Organization guidelines. The Food Frequency Questionnaire was used to obtain the intake frequency of each dietary term. Each item was scored based on consumption frequency and divided into three groups (good, intermediate, and poor) based on total dietary score. Multivariate logistic regression analysis was performed to identify dyslipidemia risk factors, as well as the joint association of PA and diet quality.

**Results:**

Dyslipidemia prevalence among urban adults in Xinjiang was 39.3%, with notable sex disparities (52.6% in males vs. 24.3% in females, *P* < 0.001). Among participants with dyslipidemia, the awareness, treatment and control rates were 6.9%, 3.1%, and 1.9%, respectively. A significant multiplicative interaction between PA and diet quality is associated with dyslipidemia (P for interaction < 0.05). Less PA and poor diet quality were associated with an increased odds of dyslipidemia. Even individuals with poor (OR = 1.464, 95% CI: 1.106–1.939) or intermediate (OR = 1.229, 95% CI: 1.003–1.505) diet quality but adhering to recommended MVPA had lower odds of dyslipidemia compared to those with good diet quality but inadequate MVPA (OR = 1.510, 95% CI: 1.252–1.821).

**Conclusions:**

Dyslipidemia prevalence was 39.3% in urban adults in Xinjiang, with limited awareness, treatment, and control. Following guideline-recommended MVPA and maintaining good diet quality were protective against dyslipidemia. Low levels of PA associated with a higher prevalence of dyslipidemia, even in individuals with good diet quality.

**Supplementary Information:**

The online version contains supplementary material available at 10.1186/s12944-024-02030-2.

## Background

Cardiovascular disease (CVD), including atherosclerotic cardiovascular disease (ASCVD), is a major long-term health concern worldwide and the leading cause of death in both urban and rural populations in China [1]. Dyslipidemia, the abnormal levels of fats in the blood, ranks as the second most significant risk factor for cardiovascular disease due to its growing prevalence [2, 3]. Managing dyslipidemia strategically is crucial in reducing the impact of CVD on health and mortality [4].

Differences in wealth, location, and lifestyle contribute to a nearly six-fold difference in the burden of CVD among Chinese provinces, and Xinjiang faces a particularly serious challenge [5]. The diverse mix of ethnic groups in Xinjiang has shaped the region's complex diet, which includes high levels of fat, carbohydrates, and meat. This unhealthy eating pattern has led to a continuous increase in cardiovascular diseases due to an imbalance in energy metabolism in the region [6–8]. Results from a 2018 prospective cohort study in Xinjiang highlight that dyslipidemia is the most common chronic disease in the area, with a prevalence rate of 34.55% [9].

The increasing prevalence of dyslipidemia is due to the rapid economic development, changing dietary habits, and sedentary lifestyles [10]. Maintaining a healthy diet and engaging in physical activity (PA) are crucial for preventing and addressing this issue [11]. Research emphasizes the importance of a balanced and diverse diet in reducing the risk of CVD and dyslipidemia [12]. Conversely, unhealthy dietary patterns and a lack of dietary diversity increase susceptibility to diseases [13, 14]. Guidelines recommend moderate-to-vigorous physical activity (MVPA) as a beneficial factor in lowering the potential risk of chronic diseases [15]. PA helps reduce plasma triglyceride levels, alleviate dyslipidemia, and enhance CVD prognosis [16]. While individual studies have explored the links between PA, diet quality, and dyslipidemia [17–19], there's a gap in understanding potential synergies between PA and diet quality in influencing dyslipidemia.

This investigation aims to examine the prevalence, awareness, treatment rates, control measures, and determinants of dyslipidemia in the urban adult population of Xinjiang. Going beyond traditional factors, we are exploring the interaction between PA and diet quality. We hypothesize that better diet quality combined with recommended MVPA may synergistically reduce the risk of dyslipidemia.

### Study population

This cross-sectional study, conducted from July 2019 to September 2021, provides reliable insights into the prevalence and factors influencing dyslipidemia among urban adults in Xinjiang. A two-stage random sampling method was used. In the first stage, a systematic sampling method was used to select two fixed communities in Urumqi in the north of Xinjiang and Korla in the south of Xinjiang. These communities were part of the Xinjiang CVD natural population cohort study. In the second stage, the sample size for each community was determined proportionally to its resident population, and eligible participants were selected using a cluster sampling method for questionnaire surveys, physical examinations, and blood sample tests. The response rate for valid questionnaires reached 95%. To be included, participants had to have resided in Xinjiang for at least 5 years. Those lacking anthropometric and biochemical data were excluded from subsequent analyses to ensure data completeness. Prior to enrollment, written informed consent was obtained from each participant in the study.

### Data collection and measurement

Baseline information was meticulously collected using a standardized and structured questionnaire, responsible by trained investigators. Prior to the main survey, the questionnaire underwent validation to ensure its accuracy and effectiveness. The detailed questionnaire covered demographic details (e.g., residence, ethnic, income, sex, education level, and age), personal lifestyle factors (e.g., smoking, PA, dietary habits), self-reported chronic diseases, and medication usage. The International Physical Activity Questionnaire (IPAQ) was used to investigate PA, including frequency and duration (days per week, duration per time), intensity of PA (vigorous, moderate intensity, and walking), and total time. The semi-quantitative food frequency questionnaire (FFQ) was used to investigate the frequency of food intake in the past year (every day, 5–6 days per week, 3–4 days per week, 1–2 days per week, less than 3 times per month, never/rarely eat) and fill in the difference questionnaire. To ensure objectivity and minimize biases, the amount of food consumed per instance was estimated using the Food Atlas of Retrospective Dietary Survey from the School of Public Health at Nanjing Medical University.

Blood pressure, weight, and height were carefully recorded, and venous blood was samples were obtained from each fasting participant. The subsequent analysis of these samples took place at the clinical laboratory of the First Affiliated Hospital of Xinjiang Medical University, utilizing a Roche cobas311 automatic biochemical detector. Various biochemical markers in plasma, including Low-density lipoprotein cholesterol (LDL-C), total cholesterol (TC), high-density lipoprotein cholesterol (HDL-C), fasting blood glucose (FBG), and triglycerides (TG), were thoroughly examined to provide a comprehensive biochemical profile.

### Definition of variables

Following Chinese guidelines for lipid management (2023) [20], high-TC, high-TG, and high-LDL-C can be defined as serum TC level ≥ 6.2 mmol/L, serum TG level ≥ 2.3 mmol/L, and serum LDL-C level ≥ 4.1 mmol/L, respectively, all of them can be defined when having lipid-lowering treatments. and low HDL-C was identified as serum HDL-C level < 1.0 mmol/L. Dyslipidemia is recognized when at least one of these abnormal lipid concentrations is present, or if a healthcare professional has diagnosed dyslipidemia. Borderline high lipid levels fall within specific thresholds: serum TC level between 5.2 and 6.2 mmol/L, serum TG level between 1.7 and 2.3 mmol/L, and serum LDL-C level between 3.4 and 4.1 mmol/L. Dyslipidemia recognition involves the disclosure of a previous diagnosis by a healthcare professional. Management includes using medications to lower blood lipid levels, and controlled dyslipidemia is achieved when participants attain TC levels below 6.2 mmol/L, TG levels below 2.3 mmol/L, or LDL-C levels below 4.1 mmol/L.

The Body Mass Index (BMI) is calculated by dividing body weight (kg) by the square of height (m^2^). Overweight and obesity are identified at BMI thresholds of ≥ 24 kg/m^2^ and ≥ 28 kg/m^2^, respectively [21]. Diabetes is established with an FBG ≥ 7.0 mmol/L or a self-reported history of diabetes [22]. Hypertension is determined by systolic blood pressure (SBP) ≥ 140 mmHg and/or diastolic blood pressure (DBP) ≥ 90 mmHg, or a hypertension self-reported history [23]. Age categories include 30–39, 40–49, 50–59, and 60 years and above. Smoking is identified in participants who report tobacco use. Education is divided into two levels: below college and college and above. Household income is divided into two groups: < 150,000 and ≥ 150,000. Ethnicity divided into two groups: Han and other (other refer to Uygur, Kazak, Hui, Mongolian, Tatar, and other ethnic minorities).

PA categorization follows established guidelines [15]: vigorous-intensity PA for 75 min/week, moderate intensity PA for 150 min/week, or an equivalent combination with MVPA. Diet quality is evaluated based on weekly grains ≥ 5 days, weekly nuts ≥ 5 days, weekly fruit ≥ 5 days, weekly seafood ≥ 1 day, weekly milk ≥ 3 days, weekly eggs ≥ 3 days, weekly red meat < 3 days, and weekly preserved food < 3 days. Each item is scored 1 or 0 based on meeting specified conditions. The total score, weighted by diet numbers, segments participants into three diet quality groups: poor (≤ 3), intermediate (4–6), and good (≥ 6) [24].

### Statistical analysis

Characteristics of study participants were examined based on whether they had dyslipidemia. Continuous variables with a normal distribution are expressed as means ± standard deviations (SDs), while those with skewed distributions are presented as medians (interquartile ranges, IQR). Categorical variables are described as counts and percentages. To compare normally distributed variables, we used the independent-sample Student t-test, and for variables with highly skewed distributions, the Mann–Whitney U test was applied. The χ^2^ test was employed to compare categorical variables.

Logistic regression models were used to identify factors associated with dyslipidemia. Variables that showed statistical significance in the univariate regression analysis were included in the multivariate analysis. These variables included sex, age, education, overweight or obesity, diabetes, hypertension, smoking, physical activity, and diet quality. The model was adjusted to minimize the impact of confounding factors. Additionally, we explored the joint association by categorizing the sample into six groups based on MVPA and diet quality, using guideline-recommended MVPA and good diet quality as reference benchmarks. Statistical analyses were conducted using the Social Sciences SPSS complex sampling function (SPSS, Chicago, Illinois, USA). The predetermined level of statistical significance was set at *P* < 0.05.

## Results

### Characteristics of participants

**Fig. 1:**
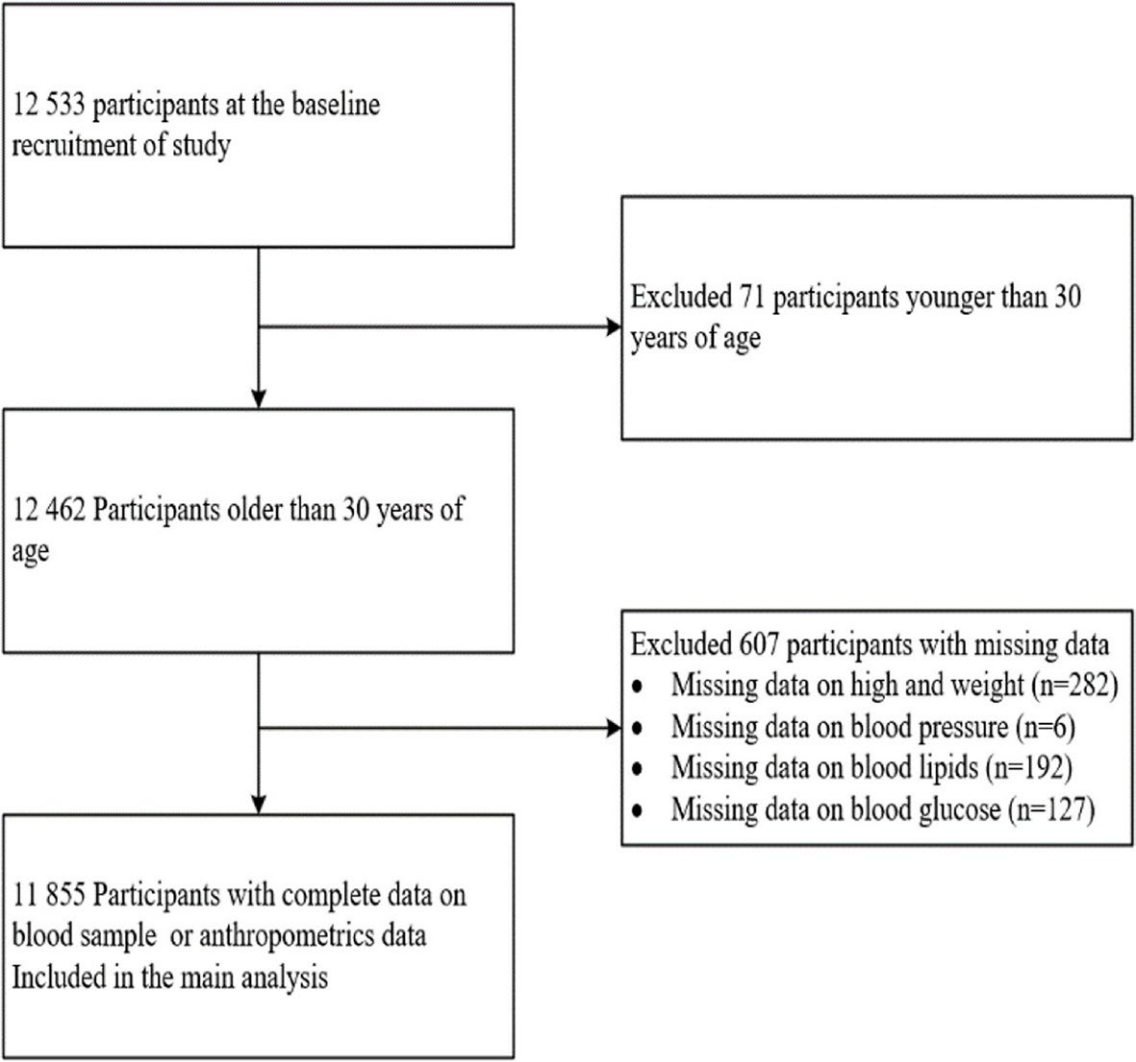
Flowchart illustrating participant enrollment

Twelve thousand five hundred thirty-three individuals were invited to participate in the study. After excluding 607 participants due to missing blood or anthropometric data and 71 individuals younger than 30 years, a total of 11,855 participants (mean age 47.05 ± 9.38 years; 53.1% men) were included in the final analysis (as shown in Fig. 1). The demographic and clinical characteristics of participants, categorized by dyslipidemia status, are detailed in Table 1. Compared to those with normal lipid levels, individuals with dyslipidemia were more likely to be male, older, have lower educational levels, and a higher prevalence of smoking. They also had a higher occurrence of overweight or obesity, hypertension, and diabetes, along with elevated levels of blood pressure, BMI, and fasting blood glucose (*P* < 0.001). However, there was no significant difference in income and ethnicity between the two groups (*P* > 0.05). Notably, overweight or obesity, hypertension, and diabetes were prevalent in 58.5%, 31.4%, and 8.1% of participants, respectively. Only 20.6% engaged in guideline-recommended moderate-to-vigorous physical activity (MVPA). Regarding diet quality, 19.1%, 54.6%, and 26.3% of participants fell into the categories of poor, intermediate, and good diet quality, respectively. Additionally, noticeable differences in MVPA levels and diet quality were observed between participants with and without dyslipidemia, with lower MVPA identified in patients with dyslipidemia. The frequency of consumption of each food category was also compared between the two groups (refer to additional Table 1).

**Table 1:** Characteristics of participants according to the presence of dyslipidemia

Variables	Total (n = 11,855)	Normal (*n* = 7194)	Dyslipidemia (*n* = 4661)	*P* value
Demographic variables				
Male, n (%)	6299 (53.1)	2986 (41.5)	3313 (71.1)	< 0.001
Age (years)	47.05 ± 9.38	46.03 ± 9.34	48.63 ± 9.22	< 0.001
30–39, n (%)	2938 (24.8)	2045 (28.4)	893 (19.2)	
40–49, n (%)	4208 (35.5)	2655 (36.9)	1553 (33.3)	
50–59, n (%)	3715 (31.3)	1974 (27.4)	1741 (37.4)	
≥ 60, n (%)	994 (8.4)	520 (7.2)	474 (10.2)	
Ethnicity, n (%)				0.821
Han	10,084 (85.1)	6115 (85.0)	3969 (85.2)	
Other	1771 (14.9)	1079 (15.0)	692 (14.8)	
college and above, n (%)	9685 (81.7)	5986 (83.2)	3699 (79.4)	< 0.001
Household income(yuan/year), n (%)				0.325
< 150,000	6369 (53.7)	3891 (54.1)	2478 (53.2)	
≥ 150,000	5486 (46.3)	3303 (45.9)	2183 (46.8)	
Chronic disease				
Hypertension, n (%)	3718 (31.4)	1753 (24.4)	1965 (42.2)	< 0.001
Diabetes, n (%)	958 (8.1)	368 (5.1)	590 (12.7)	< 0.001
Overweight/obesity, n (%)	6938 (58.5)	3427 (47.6)	3511 (75.3)	< 0.001
Physical and clinical index				
SBP (mmHg)	125.99 ± 17.60	123.00 ± 16.91	130.60 ± 17.66	< 0.001
DBP (mmHg)	79.73 ± 12.19	77.58 ± 11.62	83.04 ± 12.30	< 0.001
BMI (kg/m^2^)	24.94 ± 3.57	24.06 ± 3.37	26.30 ± 3.44	< 0.001
FBG (mmol/L)	5.32 ± 1.42	5.13 ± 1.11	5.62 ± 1.75	< 0.001
TC (mmol/L)	4.84 ± 0.99	4.59 ± 0.73	5.23 ± 1.19	< 0.001
TG (mmol/L)	1.35 (0.93 ~ 2.01)	1.07 (0.80 ~ 1.45)	2.21 (1.48 ~ 2.99)	< 0.001
LDL-C (mmol/L)	3.22 ± 0.81	2.98 ± 0.60	3.61 ± 0.94	< 0.001
HDL-C (mmol/L)	1.26 ± 0.30	1.35 ± 0.26	1.12 ± 0.29	< 0.001
Lifestyle				
Smoking, n (%)	2862 (24.1)	1203 (16.7)	1659 (35.6)	< 0.001
MVPA (min/week)	86.24 ± 215.48	93.03 ± 223.07	75.04 ± 202.80	< 0.001
Physical activity, n (%)				
Not recommended	9408 (79.4)	5597 (77.8)	3811 (81.8)	
Recommended	2447 (20.6)	1597 (22.2)	850 (18.2)	
Diet score	4.63 ± 1.33	4.72 ± 1.30	4.49 ± 1.36	< 0.001
Diet quality, n (%)				< 0.001
Poor	2259 (19.1)	1187 (16.5)	1072 (23.0)	
Intermediate	6478 (54.6)	3980 (55.3)	2498 (53.6)	
Good	3118 (26.3)	2027 (28.2)	1091 (23.4)	

The data were presented as numbers (percentages), means ± SD, or median (interquartile), as appropriate. Group differences were assessed using the X^2^ test for categorical variables, while continuous variables were analyzed using t-tests and Mann–Whitney U tests

SBP systolic blood pressure, DBP diastolic blood pressure, BMI body mass index, FBG fasting blood glucose, TC total cholesterol, TG triglyceride, LDL-C low-density lipoprotein cholesterol, HDL-C high-density lipoprotein cholesterol, MVPA moderate-to-vigorous physical activity

Physical activity: recommended was defined as vigorous intensity PA performed for 75 min/week, moderate intensity PA performed for 150 min/week, or an equivalent combination with MVPA. Diet quality: poor ≤ 3, intermediate 4–6, and good ≥ 6

The overall mean values for TC, LDL-C, and HDL-C were 4.84 mmol/L, 3.22 mmol/L, and 1.26 mmol/L, respectively, while the median TG level was 1.35 mmol/L. Men exhibited higher mean values of TC, LDL-C, and median TG but lower HDL-C than their female counterparts. The mean TC values demonstrated an increasing trend with age, while the means of LDL-C and the median TG increased with age until participants reached their 60 s before experiencing a decline. Conversely, HDL-C exhibited a gradual decrease after participants reached their 50 s. Individuals with lower educational levels, hypertension, diabetes, overweight or obesity, a smoking habit, low MVPA engagement, and poor diet quality were predisposed to higher TC, TG, and LDL-C, and lower HDL-C, in comparison to their counterparts (all *P* < 0.05, refer to Additional Table 2 and Additional Fig. 1).

### Prevalence of dyslipidemia

The overall prevalence of dyslipidemia was 39.3%, with distinct rates for high TC, TG, LDL-C, and low HDL-C at 9.8%, 19.6%, 14.9%, and 17.5%, respectively. There was a noticeable difference between males and females, with a higher prevalence in males (52.6% vs. 24.3%, *P* < 0.001). Dyslipidemia prevalence increased with age, reaching the highest at 47.7% in the ≥ 60 age group. High TC and LDL-C followed a similar trend, peaking at 18.4% and 22.3%, respectively. The highest prevalence of high TG was observed in the 50–59 age group at 23.3% (*P* < 0.001), while no age-based variation was noted for low HDL-C (*P* > 0.05). Participants with overweight or obesity, diabetes, hypertension, and a history of smoking had higher dyslipidemia prevalence and elevated lipid levels (*P* < 0.001). Engaging in guideline-recommended MVPA was associated with lower dyslipidemia prevalence (34.7% vs. 40.5%, *P* < 0.001). Dyslipidemia prevalence also varied with diet quality, with rates of 47.5%, 38.6%, and 35.0% for poor, intermediate, and good diet quality, respectively (*P* < 0.001, refer to Additional Table 3).

Exploring the prevalence of high and borderline high lipid subtypes across sexes revealed that 24.4%, 15.9%, and 25.6% of all participants had borderline high TC, TG, and LDL-C, respectively. Males exhibited prevalence rates of 25.8%, 19.6%, and 28.2% for borderline high TC, TG, and LDL-C, while females demonstrated rates of 22.7%, 11.7%, and 22.7% for the corresponding lipid subtypes (refer to Additional Fig. 2).

### Awareness, treatment, and control of dyslipidemia

**Fig. 2:**
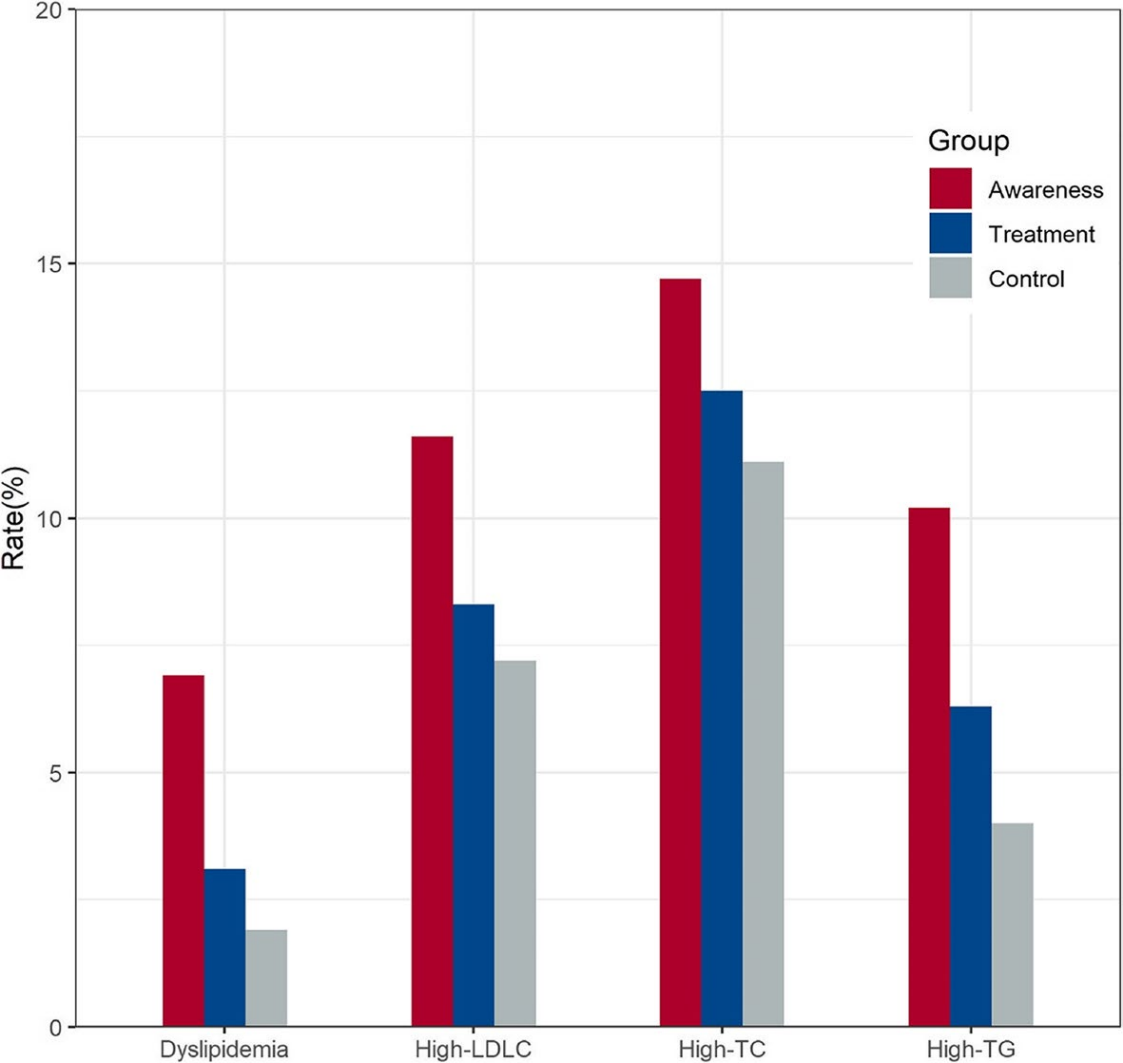
Awareness, treatment, and control rates of dyslipidemia, and lipid subtypes in Xinjiang

Among participants with dyslipidemia, the rates of awareness, treatment, and control of dyslipidemia were notably modest at 6.9%, 3.1%, and 1.9%, respectively. Delving into participants with elevated TC concentrations (≥ 6.2 mmol/L) or those reporting the use of cholesterol-lowering medications, merely 14.7% were aware of their condition, 12.5% underwent treatment, and a mere 11.1% attained a TC concentration < 6.2 mmol/L. For individuals with elevated TG concentrations (≥ 2.3 mmol/L) or using cholesterol-lowering medications, awareness, intervention, and management rates were 10.2%, 6.3%, and 4.0%, respectively. Participants with elevated LDL-C concentrations (≥ 4.1 mmol/L) or using cholesterol-lowering medications exhibited awareness, treatment, and control rates at 11.6%, 8.3%, and 7.2%, respectively (refer to Fig. 2).

### Factors associated with dyslipidemia

**Fig. 3:**
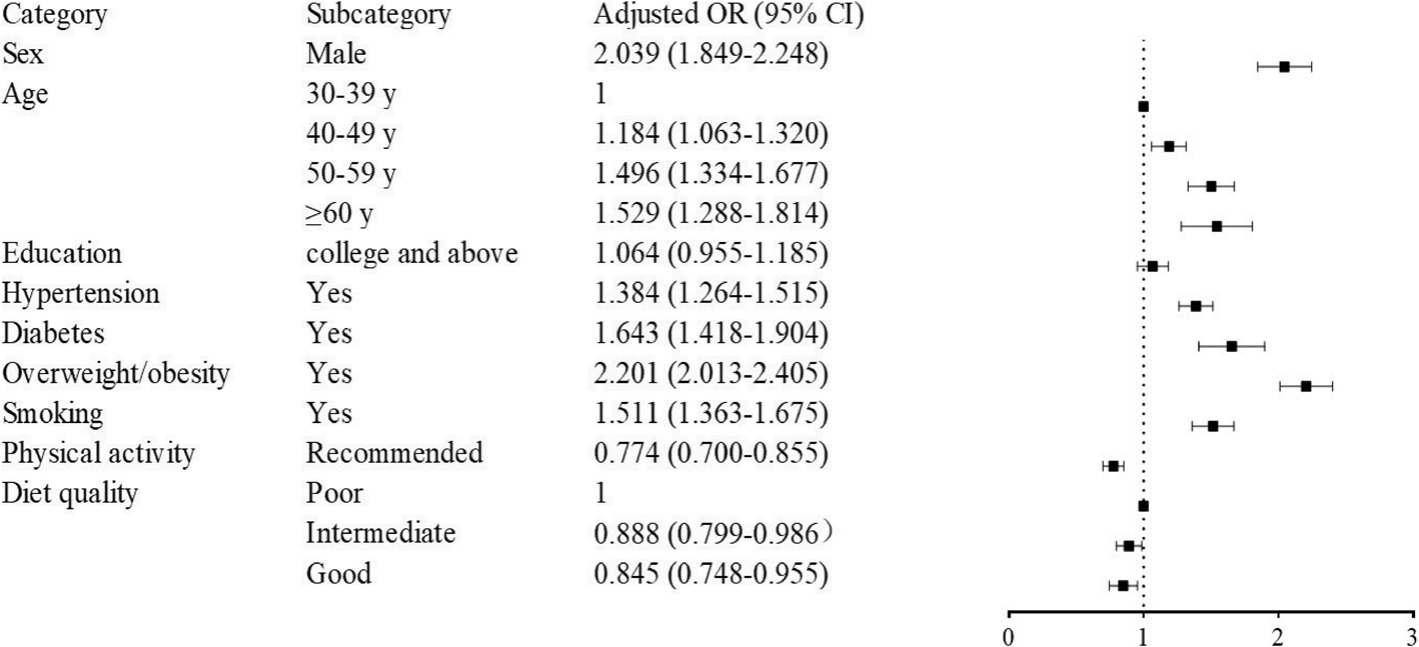
Multivariable logistic regression explored the risk factors of dyslipidemia. The model was adjusted for sex, age, education level, overweight or obesity, diabetes, hypertension, smoking, physical activity, and diet quality

Multivariable logistic regression reveals that males demonstrate an elevated predisposition to dyslipidemia than females (OR = 2.039, 95% CI: 1.849–2.248). Age stratification into four groups shows an increasing risk with age, particularly in the ≥ 60 years category (OR = 1.529, 95% CI: 1.288–1.814). Dyslipidemia is more prevalent among those who are overweight or obese (OR = 2.201, 95% CI: 2.013–2.405), have diabetes (OR = 1.643, 95% CI: 1.418–1.904), and hypertension (OR = 1.384, 95% CI: 1.264–1.515) compared to individuals without these chronic conditions. Smoking is identified as a significant risk factor for dyslipidemia (OR = 1.511, 95% CI: 1.363–1.675). Conversely, engaging in guideline-recommended moderate-to-vigorous physical activity (MVPA) (OR = 0.774, 95% CI: 0.700–0.855), having an intermediate diet quality (OR = 0.888, 95% CI: 0.799–0.986), and having good diet quality (OR = 0.845, 95% CI: 0.748–0.955) show an inverse correlation with dyslipidemia (depicted in Fig. 3).

When exploring the potential roles of PA and diet quality in dyslipidemia, an examination of dietary scores and MVPA amounts reveals non-linear dose–response patterns in their association with a reduced risk of dyslipidemia (refer to Additional Fig. 3).

### Joint associations of PA and diet quality with dyslipidemia

**Fig. 4:**
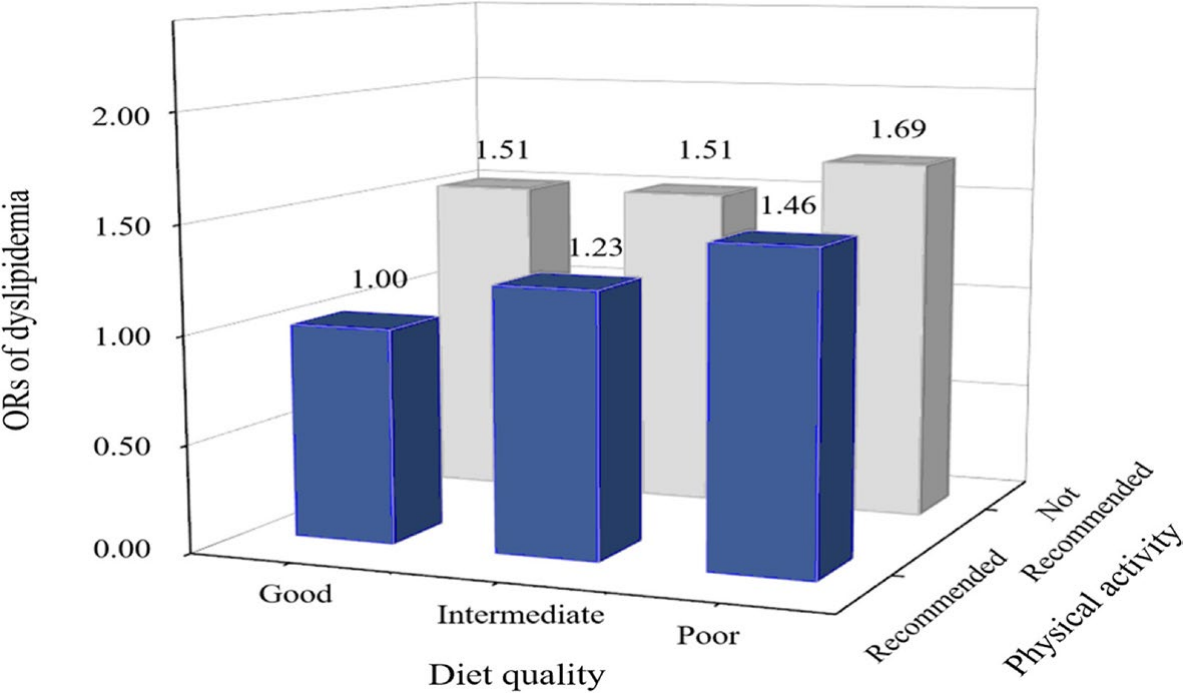
Joint associations of physical activity and diet quality with dyslipidemia. The multivariable logistic regression model was adjusted for sex, age, overweight or obesity, diabetes, hypertension, smoking, and education level. The *P* for interaction is < 0.001

The analysis of interaction revealed a noteworthy interaction between PA level and diet quality (*P* < 0.05). By cross-classifying participants based on recommended MVPA and diet quality, adjusted regression analysis showed that, compared with those who engaged in recommended MVPA and maintained good diet quality, those who did not receive MVPA and had poor diet quality were associated with an increased odds for dyslipidemia (OR = 1.692, 95%CI: 1.398–2.048). In the guideline-recommended MVPA group, poor diet quality (OR = 1.464, 95%CI: 1.106–1.939) and moderate diet quality (OR = 1.229, 95%CI: 1.003–1.505) had a lower OR for dyslipidemia than those who had a good diet but did not engage in recommended MVPA (OR = 1.510, 95% CI: 1.252–1.821) (see Fig. 4 and Additional Table 4).

### Joint associations of PA and age, diet quality, and age with dyslipidemia

Interactions between physical activity and age, as well as diet quality and age, were also examined. Participants were categorized based on their physical activity level and age, with the reference group being 30–39 years old individuals engaged in guideline-recommended MVPA. Adjusted regression analysis revealed that participants meeting MVPA levels had a lower OR for dyslipidemia compared to participants in the same age group who did not meet the recommended physical activity levels (as shown in Additional Table 5). For the interaction between diet quality and age, when stratified by diet quality, higher age was associated with a higher prevalence of dyslipidemia within each diet quality group (as shown in Additional Table 6).

## Discussion

In recent times, the prevalence of dyslipidemia in China has not only remained high but has also shown a continuous increase. The 2002 Chinese National Nutrition and Health Survey reported a dyslipidemia prevalence of 18.6% among Chinese adults [25]. However, the subsequent national survey in 2018 revealed a substantial surge, with the overall prevalence soaring to 35.6% [20]. Understanding the complex epidemiology and identifying influential factors of dyslipidemia is crucial in public health to tackle the growing burden of CVD. This study provides an updated assessment of the lipid profile in Xinjiang. In this cross-sectional study, the prevalence of dyslipidemia in the population was 39.3%, significantly higher than reported in Xinjiang and other regions of China [9, 26–28]. Despite this high prevalence, awareness, treatment, and control rates of dyslipidemia stood at 6.9%, 3.1%, and 1.9%, respectively. The combination of rapid economic development, an aging population, and lifestyle changes are major contributors to the increasing prevalence of dyslipidemia [10]. Moreover, while high TC, TG, and LDL-C prevalence within the study population was reported at 9.8%, 19.6%, and 14.9%, respectively, borderline high TC, TG, and LDL-C prevalence substantially presented higher, registering at 24.4%, 15.9%, and 25.6%. This emphasizes the need for prompt intervention and control measures; otherwise, the prevalence of dyslipidemia is poised for further escalation in the foreseeable future.

Our findings reveal a noticeable difference in dyslipidemia prevalence between sexes, with males having a higher incidence compared to females. This observed gap can be attributed to entrenched unhealthy lifestyle behaviors more prevalent among males, such as smoking, increased energy intake, and reduced physical activity levels [29, 30]. Compared with males, females have a lower prevalence of dyslipidemia, which may be linked to the protective effect of estrogen in women [31, 32]. Additionally, there is a significant increase in dyslipidemia prevalence with advancing age, where age plays an undeniable role as an independent risk factor for dyslipidemia. This age-related rise is partly due to decreased metabolism and physical performance in the elderly, combined with an increased burden of other health conditions. The study highlights a higher susceptibility to dyslipidemia among participants with underlying chronic conditions, such as overweight or obesity, diabetes, and hypertension, aligning with existing literature [26, 33]. This emphasizes the need for targeted preventive and control measures, focusing on intensified efforts among males, the elderly demographic, and individuals dealing with chronic non-communicable diseases.

Dyslipidemia, a complex health concern, intricately links with diet quality and lifestyle practices. A wealth of studies supports the protective influence of regular PA and health-conscious dietary habits against the onset of dyslipidemia [11, 34, 35]. Our empirical findings affirm this concept, showing that adherence to guideline-recommended MVPA and adopting a high-quality diet are inversely linked to the prevalence of dyslipidemia. A nuanced exploration into the joint association of recommended MVPA and diet quality reveals that the group aligning with recommended MVPA and good diet quality exhibits the most robust protection against dyslipidemia among all cohorts. The beneficial effects of PA on blood lipids unfold across several dimensions. Firstly, PA elevates the body's metabolic rate, facilitating the breakdown of fats and dissipating excess body heat to prevent its conversion into adipose tissue. Secondly, during prolonged low-intensity exercise, fat becomes a crucial energy source through the hydrolysis of TG. Thirdly, the increase in mitochondrial density facilitated by physical activity creates an optimal environment for the β-oxidation of fats [36–38]. It's noteworthy that there's a lack of targeted pharmaceutical interventions for HDL-C. However, consistent engagement in physical activity, maintaining an ideal body weight, and quitting smoking emerge as viable approaches to gradually enhance HDL-C levels [39]. In the aspect of primary prevention, particularly for low-risk populations, and as a supplementary content to pharmaceutical interventions for high-risk groups, fostering a health-conscious lifestyle coupled with regular PA assumes pivotal significance [40]. It's essential to highlight that, even in our busy lives where avoiding fast food is tough, regular physical activity helps offset the negative impact of fast-food consumption on health, including dyslipidemia.

### Strengths and limitations

This cross-sectional study in Xinjiang comprehensively captures a large and diverse adult population, using standardized tools and rigorous data collection methods. However, there are some limitations to consider: Firstly, being a cross-sectional study, it only reveals correlations, and causation cannot be established. Secondly, the impact of additional factors like sedentary behavior and alcohol consumption on dyslipidemia was not explored. Thirdly, self-reported data on smoking, physical activity, and dietary habits may be prone to recall bias. Lastly, diabetes was defined based on fasting plasma glucose without additional glucose tolerance testing, potentially leading to variations in diabetes prevalence.

## Conclusions

A rising occurrence of dyslipidemia in Xinjiang's adult population is coupled with notably low rates of awareness, treatment, and control. Swift intervention strategies are essential, especially targeting high-risk groups like the elderly, individuals with chronic diseases, and those embracing unhealthy lifestyles. Effective educational campaigns and intensified management protocols play a crucial role. Additionally, the study highlights the protective effects of maintaining a good diet and following recommended physical activity guidelines in reducing the risk of dyslipidemia. Conversely, insufficient physical activity appears linked to a higher dyslipidemia prevalence, even among individuals with a healthy diet. Hence, promoting health-focused lifestyles is of utmost importance, complementing clinical approaches to curb dyslipidemia. This comprehensive strategy integrates preventive healthcare with clinical methods for a more impactful prevention and reduction of dyslipidemia.

### Supplementary Information

**Additional file 1: Additional table 1. **Frequency of each dietary term in dyslipidemia and normal groups. **Additional table 2. **Lipid levels based on different subpopulation. **Additional Fig. 1.** Kernel density plots for lipid subtypes based on age, sex, and physical activity. **Additional table 3. **Prevalence of different types of dyslipidemia. **Additional Fig. 2.** The prevalence of borderline high and high of lipid subtypes based on sex. **Additional Fig. 3.** Dose-response association between moderate-to-vigorous physical activity, diet quality with dyslipidemia. **Additional table 4. **Joint associations of physical activity and diet quality with dyslipidemia. **Additional table 5. **Joint associations of physical activity and age with dyslipidemia. **Additional table 6. **Joint associations of diet quality and age with dyslipidemia.

## Data Availability

No datasets were generated or analysed during the current study.

## Abbreviations

ASCVD: Atherosclerotic cardiovascular disease
BMI: Body mass index
CVD: Cardiovascular disease
DBP: Diastolic blood pressure
FBG: Fasting blood glucose
HDL-C: High-density lipoprotein cholesterol
IPAQ: International Physical Activity Questionnaire
IQR: Interquartile ranges
LDL-C: Low-density lipoprotein cholesterol
MVPA: Moderate-to-vigorous physical activity
OR: Odds ratio
PA: Physical activity
SBP: Systolic blood pressure
SDs: Standard deviations
TC: Total cholesterol
TG: Triglycerides
95% CI: 95% Confidence interval

## References

[1] Zhao D, Liu J, Wang M, Zhang X, Zhou M (2019). Epidemiology of cardiovascular disease in China: current features and implications. Nat Rev Cardiol.

[2] Yang G, Wang Y, Zeng Y, Gao GF, Liang X, Zhou M, Wan X, Yu S, Jiang Y, Naghavi M (2013). Rapid health transition in China, 1990–2010: findings from the Global Burden of Disease Study 2010. Lancet.

[3] Zhou M, Wang H, Zhu J, Chen W, Wang L, Liu S, Li Y, Wang L, Liu Y, Yin P (2016). Cause-specific mortality for 240 causes in China during 1990–2013: a systematic subnational analysis for the Global Burden of Disease Study 2013. Lancet.

[4] Ference BA, Ginsberg HN, Graham I, Ray KK, Packard CJ, Bruckert E, Hegele RA, Krauss RM, Raal FJ, Schunkert H (2017). Low-density lipoproteins cause atherosclerotic cardiovascular disease. 1. Evidence from genetic, epidemiologic, and clinical studies. A consensus statement from the European Atherosclerosis Society Consensus Panel. Eur Heart J.

[5] Liu S, Li Y, Zeng X, Wang H, Yin P, Wang L, Liu Y, Liu J, Qi J, Ran S (2019). Burden of Cardiovascular Diseases in China, 1990–2016: Findings From the 2016 Global Burden of Disease Study. JAMA Cardiol.

[6] Hailili G, Chen Z, Tian T, Fu W-H, Pei H-L, Mahan Y, Luo T, Alimu D, Wang L, Zhang G-Z (2020). Dietary patterns and their associations with the metabolic syndrome and predicted 10-year risk of CVD in northwest Chinese adults. Br J Nutr.

[7] Wang WQ, Wei B, Song YP, Guo H, Zhang XH, Wang XP, Yan YZ, Ma JL, Wang K, Keerman M (2021). Metabolically healthy obesity and unhealthy normal weight rural adults in Xinjiang: prevalence and the associated factors. BMC Public Health.

[8] Guo H, Gao X, Ma R, Liu J, Ding Y, Zhang M, Zhang J, Mu L, He J, Yan Y (2017). Prevalence of Metabolic Syndrome and its Associated Factors among Multi-ethnic Adults in Rural Areas in Xinjiang, China. Sci Rep.

[9] Tao L, Tian T, Liu L, Zhang Z, Sun Q, Sun G, Dai J, Yan H (2022). Cohort profile: The Xinjiang Multiethnic Cohort (XMC) study. BMJ Open.

[10] Pirillo A, Casula M, Olmastroni E, Norata GD, Catapano AL (2021). Global epidemiology of dyslipidaemias. Nat Rev Cardiol.

[11] Mach F, Baigent C, Catapano AL, Koskinas KC, Casula M, Badimon L, Chapman MJ, De Backer GG, Delgado V, Ference BA (2020). 2019 ESC/EAS Guidelines for the management of dyslipidaemias: lipid modification to reduce cardiovascular risk. Eur Heart J.

[12] Trautwein EA, McKay S. The Role of Specific Components of a Plant-Based Diet in Management of Dyslipidemia and the Impact on Cardiovascular Risk. Nutrients. 2020;12(9):10. 10.3390/nu12092671 PMC7551487 32883047

[13] Ferguson CC, Knol LL, Ellis AC (2021). Visceral adiposity index and its association with Dietary Approaches to Stop Hypertension (DASH) diet scores among older adults: National Health and Nutrition Examination Surveys 2011–2014. Clin Nutr.

[14] Trichopoulou A, Costacou T, Bamia C, Trichopoulos D (2003). Adherence to a Mediterranean diet and survival in a Greek population. N Engl J Med.

[15] Bull FC, Al-Ansari SS, Biddle S, Borodulin K, Buman MP, Cardon G, Carty C, Chaput JP, Chastin S, Chou R (2020). World Health Organization 2020 guidelines on physical activity and sedentary behaviour. Br J Sports Med.

[16] Tian D, Meng J (2019). Exercise for Prevention and Relief of Cardiovascular Disease: Prognoses, Mechanisms, and Approaches. Oxid Med Cell Longev.

[17] Lee K, Kim H, Rebholz CM, Kim J. Association between Different Types of Plant-Based Diets and Risk of Dyslipidemia: A Prospective Cohort Study. Nutrients. 2021;13(1):5-6. 10.3390/nu13010220 PMC7828805 33466664

[18] Sonestedt E, Hellstrand S, Drake I, Schulz CA, Ericson U, Hlebowicz J, Persson MM, Gullberg B, Hedblad B, Engstrom G, et al. Diet Quality and Change in Blood Lipids during 16 Years of Follow-up and Their Interaction with Genetic Risk for Dyslipidemia. Nutrients. 2016;8(5):7-8. 10.3390/nu8050274 PMC4882687 27171109

[19] Du L, Hong F, Luo P, Wang Z, Zeng Q, Guan H, Liu H, Yuan Z, Xu D, Nie F (2022). Patterns and demographic correlates of domain-specific physical activities and their associations with dyslipidaemia in China: a multiethnic cohort study. BMJ Open.

[20] Joint Committee on the Chinese Guidelines for Lipid M (2023). Chinese guidelines for lipid management (2023). Zhonghua Xin Xue Guan Bing Za Zhi.

[21] Zeng Q, Li N, Pan XF, Chen L, Pan A (2021). Clinical management and treatment of obesity in China. Lancet Diabetes Endocrinol.

[22] American Diabetes A (2013). Diagnosis and classification of diabetes mellitus. Diabetes Care.

[23] Williams B, Mancia G, Spiering W, Rosei EA, Azizi M, Burnier M, Clement DL, Coca A, de Simone G, Dominiczak A (2019). 2018 ESC/ESH Guidelines for the management of arterial hypertension. Kardiol Pol.

[24] Alvarez-Alvarez I, de Rojas JP, Fernandez-Montero A, Zazpe I, Ruiz-Canela M, Hidalgo-Santamaria M, Bes-Rastrollo M, Martinez-Gonzalez MA (2018). Strong inverse associations of Mediterranean diet, physical activity and their combination with cardiovascular disease: The Seguimiento Universidad de Navarra (SUN) cohort. Eur J Prev Cardiol.

[25] Zhao WH, Zhang J, You Y, Man QQ, Li H, Wang CR, Zhai Y, Li Y, Jin SG, Yang XG (2005). Epidemiologic characteristics of dyslipidemia in people aged 18 years and over in China. Zhonghua Yu Fang Yi Xue Za Zhi.

[26] Pan L, Yang Z, Wu Y, Yin RX, Liao Y, Wang J, Gao B, Zhang L (2016). China National Survey of Chronic Kidney Disease Working G: The prevalence, awareness, treatment and control of dyslipidemia among adults in China. Atherosclerosis.

[27] Xing L, Jing L, Tian Y, Yan H, Zhang B, Sun Q, Dai D, Shi L, Liu D, Yang Z (2020). Epidemiology of dyslipidemia and associated cardiovascular risk factors in northeast China: A cross-sectional study. Nutr Metab Cardiovasc Dis.

[28] Qiu L, Wang W, Sa R, Liu F (2021). Prevalence and Risk Factors of Hypertension, Diabetes, and Dyslipidemia among Adults in Northwest China. Int J Hypertens.

[29] Shi J, Bai Y, Qiu S, Li Y, Kou C, Tao Y, Zhen Q, Gu Y, Yu Y, Zhang K (2018). Classified status of smoking and quitting has different associations with dyslipidemia in residents in northeast China. Clin Chim Acta.

[30] Li L, Ouyang F, He J, Qiu D, Luo D, Xiao S (2022). Associations of Socioeconomic Status and Healthy Lifestyle With Incidence of Dyslipidemia: A Prospective Chinese Governmental Employee Cohort Study. Front Public Health.

[31] Ambikairajah A, Walsh E, Cherbuin N (2019). Lipid profile differences during menopause: a review with meta-analysis. Menopause.

[32] Ko SH, Kim HS. Menopause-Associated Lipid Metabolic Disorders and Foods Beneficial for Postmenopausal Women. Nutrients. 2020;12(1):2-3. 10.3390/nu12010202 PMC7019719 31941004

[33] Zhang FL, Xing YQ, Wu YH, Liu HY, Luo Y, Sun MS, Guo ZN, Yang Y (2017). The prevalence, awareness, treatment, and control of dyslipidemia in northeast China: a population-based cross-sectional survey. Lipids Health Dis.

[34] Ferraro RA, Fischer NM, Xun H, Michos ED (2020). Nutrition and physical activity recommendations from the United States and European cardiovascular guidelines: a comparative review. Curr Opin Cardiol.

[35] Force USPST, Krist AH, Davidson KW, Mangione CM, Barry MJ, Cabana M, Caughey AB, Donahue K, Doubeni CA, Epling JW (2020). Behavioral Counseling Interventions to Promote a Healthy Diet and Physical Activity for Cardiovascular Disease Prevention in Adults With Cardiovascular Risk Factors: US Preventive Services Task Force Recommendation Statement. JAMA..

[36] Meyer-Lindemann U, Moggio A, Dutsch A, Kessler T, Sager HB. The Impact of Exercise on Immunity, Metabolism, and Atherosclerosis. Int J Mol Sci. 2023;24(4):9-11. 10.3390/ijms24043394 PMC9967592 36834808

[37] Valenzuela PL, Ruilope LM, Santos-Lozano A, Wilhelm M, Krankel N, Fiuza-Luces C, Lucia A (2023). Exercise benefits in cardiovascular diseases: from mechanisms to clinical implementation. Eur Heart J.

[38] Nasi M, Patrizi G, Pizzi C, Landolfo M, Boriani G, Dei Cas A, Cicero AFG, Fogacci F, Rapezzi C, Sisca G (2019). The role of physical activity in individuals with cardiovascular risk factors: an opinion paper from Italian Society of Cardiology-Emilia Romagna-Marche and SIC-Sport. J Cardiovasc Med (Hagerstown).

[39] Berberich AJ, Hegele RA (2022). A Modern Approach to Dyslipidemia. Endocr Rev.

[40] Barone Gibbs B, Hivert MF, Jerome GJ, Kraus WE, Rosenkranz SK, Schorr EN, Spartano NL, Lobelo F, Cardiometabolic H, American Heart Association Council on L (2021). Physical Activity as a Critical Component of First-Line Treatment for Elevated Blood Pressure or Cholesterol: Who, What, and How?: A Scientific Statement From the American Heart Association. Hypertension.

