# Supplementary material for: Joint association of physical activity and diet quality with dyslipidemia: a cross-sectional study in Western China
Source: Lipids Health Dis. 2024 Feb 10;23:46. doi: 10.1186/s12944-024-02030-2 (PMC10858468; doi:10.1186/s12944-024-02030-2)
Supplement: Supplementary file 1 — Additional file 1: Additional table 1. Frequency of each dietary term in dyslipidemia and normal groups. Additional table 2. Lipid levels based on different subpopulation. Additional Fig. 1. Kernel density plots for lipid subtypes based on age, sex, and physical activity. Additional table 3. Prevalence of different types of dyslipidemia. Additional Fig. 2. The prevalence of borderline high and high of lipid subtypes based on sex. Additional Fig. 3. Dose-response association between moderate-to-vigorous physical activity, diet quality with dyslipidemia. Additional table 4. Joint associations of physical activity and diet quality with dyslipidemia. Additional table 5. Joint associations of physical activity and age with dyslipidemia. Additional table 6. Joint associations of diet quality and age with dyslipidemia. [file 12944_2024_2030_MOESM1_ESM.docx]

**Supplementary materials**

**Joint Association of Physical Activity and Diet Quality with Dyslipidemia: A Cross-Sectional Study in Western China**

Munire Mutalifu^1*^, Qian Zhao^1*^, Ying Wang^1^, Xieyire Hamulati^1^, Yu-Shan Wang^3^, Lei Deng^4^, Niyaziaili Adili^1^, Fen Liu^1^, Yi-Ning Yang^1,2†^, Xiao-Mei Li^1†^

^1^State Key Laboratory of Pathogenesis, Prevention and Treatment of High Incidence Diseases in Central Asia, Department of Cardiology, First Affiliated Hospital of Xinjiang Medical University, Urumqi, China.

^2^Department of Cardiology, People’s Hospital of Xinjiang Uygur Autonomous Region, Urumqi, China.

^3^Center of Health Management, The First Affiliated Hospital of Xinjiang Medical University, Urumqi, China.

^4^Baoshihua Korla Hospital, Korla, China.

^†^Corresponding author: 1^st^ ，Prof. Xiao-Mei Li; 2^nd^Prof. Yi-Ning Yang

^*^Contribute equally to this study

Prof. Xiao-Mei Li, State Key Laboratory of Pathogenesis, Prevention and Treatment of High Incidence Diseases in Central Asia, Department of Cardiology, First Affiliated Hospital of Xinjiang Medical University; and Xinjiang Key Laboratory of Cardiovascular Disease Research, Clinical Medical Research Institute of Xinjiang Medical University, Urumqi, China. Email: Tel: 13079926866, Fax: 730718, Email: [lixm505@163.com](mailto:lixm505@163.com)

Prof. Yi-Ning Yang, PhD, Department of Cardiology, People’s Hospital of Xinjiang Uygur Autonomous Region; State Key Laboratory of Pathogenesis, Prevention and Treatment of High Incidence Diseases in Central Asia, Department of Cardiology, First Affiliated Hospital of Xinjiang Medical University; and Xinjiang Key Laboratory of Cardiovascular Disease Research, Clinical Medical Research Institute of Xinjiang Medical University, Urumqi, China. Email: [yangyn5126@163.com](mailto:yangyn5126@163.com).

**Additional table 1.** Frequency of each dietary term in dyslipidemia and normal groups

| **Variables** | **Normal (n=7194)** | **Dyslipidemia (n=4661)** | ***P* value** |
| --- | --- | --- | --- |
| Weekly grains |  |  | < 0.001 |
| ≥5 days | 2142 (29.8) | 1240 (26.6) |  |
| < days | 5052 (70.2) | 3421 (73.4) |  |
| Weekly nuts |  |  | 0.499 |
| ≥5 days | 2015 (28.0) | 1279 (27.4) |  |
| < 5 days | 5179 (72.0) | 3382 (72.6) |  |
| Weekly fruit |  |  | < 0.001 |
| ≥5 days | 5974 (83.0) | 3600 (77.2) |  |
| < 5 days | 1220 (17.0) | 1061 (22.8) |  |
| Weekly seafood |  |  | 0.009 |
| ≥1 day | 4280 (59.5) | 2600 (57.1) |  |
| < 1 day | 2914 (40.5) | 2001 (42.9) |  |
| Weekly milk |  |  | < 0.001 |
| ≥3 days | 5539 (74.2) | 3237 (69.4) |  |
| < 3 days | 1855 (25.8) | 1424 (30.6) |  |
| Weekly eggs |  |  | 0.275 |
| ≥3 days | 6143 (85.4) | 3946 (84.7) |  |
| < 3 days | 1051 (14.6) | 715 (15.3) |  |
| Weekly red meat |  |  | < 0.001 |
| ≥3 days | 5511 (76.6) | 3716 (79.7) |  |
| < 3 days | 1683 (23.4) | 945 (20.3) |  |
| Weekly preserved food |  |  | < 0.001 |
| ≥3 days | 791 (11.0) | 622 (13.3) |  |
| < 3 days | 6403 (89.0) | 4039 (86.7) |  |

**Additional table 2.** Lipid levels based on different subpopulation

| **Variables** | **TC** (mmol/L) | **TG (**mmol/L) | **LDL-C** (mmol/L) | **HDL-C** (mmol/L) |
| --- | --- | --- | --- | --- |
| Total | 4.84 ± 0.99 | 1.35 (0.93~2.01) | 3.22 ± 0.81 | 1.26 ± 0.30 |
| Sex |  |  |  |  |
| Male | 4.88 ± 1.02 | 1.64 (1.16~2.43) | 3.31 ± 0.83 | 1.17 ± 0.26 |
| Female | 4.80 ± 0.96 | 1.08 (0.79~1.55) | 3.13 ±0.78 | 1.37 ± 0.30 |
| *P* value | <0.001 | <0.001 | <0.001 | <0.001 |
| Age, (years) |  |  |  |  |
| 30-39 | 4.57 ± 0.91 | 1.17 (0.80~1.79) | 2.99 ± 0.75 | 1.25 ± 0.28 |
| 40-49 | 4.82 ± 0.92 | 1.33 (0.91~2.02) | 3.22 ± 0.78 | 1.27 ± 0.30 |
| 50-59 | 5.01 ± 1.03 | 1.48 (1.05~2.16) | 3.38 ± 0.84 | 1.26 ± 0.31 |
| ≥60 | 5.09 ± 1.16 | 1.46 (1.05~2.00) | 3.34 ± 0.88 | 1.24 ± 0.28 |
| *P* value | <0.001 | <0.001 | <0.001 | 0.001 |
| Education |  |  |  |  |
| college below | 4.89 ± 1.04 | 1.46 (1.03~2.14) | 3.28 ± 0.83 | 1.23 ± 0.29 |
| college and above | 4.83 ± 0.98 | 1.33 (0.91~1.97) | 3.21 ± 0.81 | 1.27 ± 0.30 |
| *P* value | 0.03 | <0.001 | <0.001 | <0.001 |
| Hypertension |  |  |  |  |
| Yes | 4.96 ± 1.08 | 1.65 (1.15~2.44) | 3.33 ± 0.87 | 1.20 ± 0.28 |
| No | 4.79 ± 0.95 | 1.23 (0.86~1.82) | 3.18 ± 0.78 | 1.29 ± 0.30 |
| *P* value | <0.001 | <0.001 | <0.001 | <0.001 |
| Diabetes |  |  |  |  |
| Yes | 4.92 ± 1.23 | 1.80 (1.25~2.80) | 3.28 ± 0.93 | 1.15 ± 0.27 |
| No | 4.84 ± 0.97 | 1.32 (0.91~1.95) | 3.22 ± 0.80 | 1.27 ± 0.30 |
| *P* value | 0.03 | <0.001 | 0.06 | <0.001 |
| Overweight/obesity |  |  |  |  |
| Yes | 4.93 ± 1.02 | 1.61 (1.12~2.35) | 3.33 ± 0.83 | 1.17 ± 0.25 |
| No | 4.72 ± 0.93 | 1.06 (0.78~1.51) | 3.08 ± 0.77 | 1.38 ± 0.31 |
| *P* value | <0.001 | <0.001 | <0.001 | <0.001 |
| Smoking |  |  |  |  |
| Yes | 4.95 ± 1.01 | 1.78 (1.26~2.64) | 3.36 ± 0.83 | 1.14 ± 0.25 |
| No | 4.81 ± 0.98 | 1.23 (0.87~1.83) | 3.18 ± 0.80 | 1.30 ± 0.30 |
| *P* value | <0.001 | <0.001 | <0.001 | <0.001 |
| Physical activity |  |  |  |  |
| Recommended | 4.80 ± 0.95 | 1.30 (0.90~1.89) | 3.21 ± 0.81 | 1.31 ± 0.31 |
| Not recommended | 4.85 ± 1.00 | 1.37 (0.94~2.05) | 3.23 ± 0.81 | 1.25 ± 0.29 |
| *P* value | 0.007 | <0.001 | 0.22 | <0.001 |
| Diet quality |  |  |  |  |
| Poor | 4.90 ± 1.01 | 1.53 (1.03~2.32) | 3.29 ± 0.83 | 1.21 ± 0.28 |
| Intermediate | 4.83 ± 0.99 | 1.34 (0.93~1.98) | 3.21 ± 0.81 | 1.25 ± 0.29 |
| Good | 4.83 ± 0.97 | 1.25 (0.87~1.86) | 3.21 ± 0.80 | 1.31 ± 0.31 |
| *P* value | 0.004 | <0.001 | <0.001 | <0.001 |

Data were shown as number (percentages), means ± SD or median (interquartile), as appropriate. Differences between the groups were examined by *t* test and Mann-Whitney U test for continuous variables

TC total cholesterol, TG triglyceride, LDL-C low-density lipoprotein cholesterol, HDL-C high-density lipoprotein cholesterol


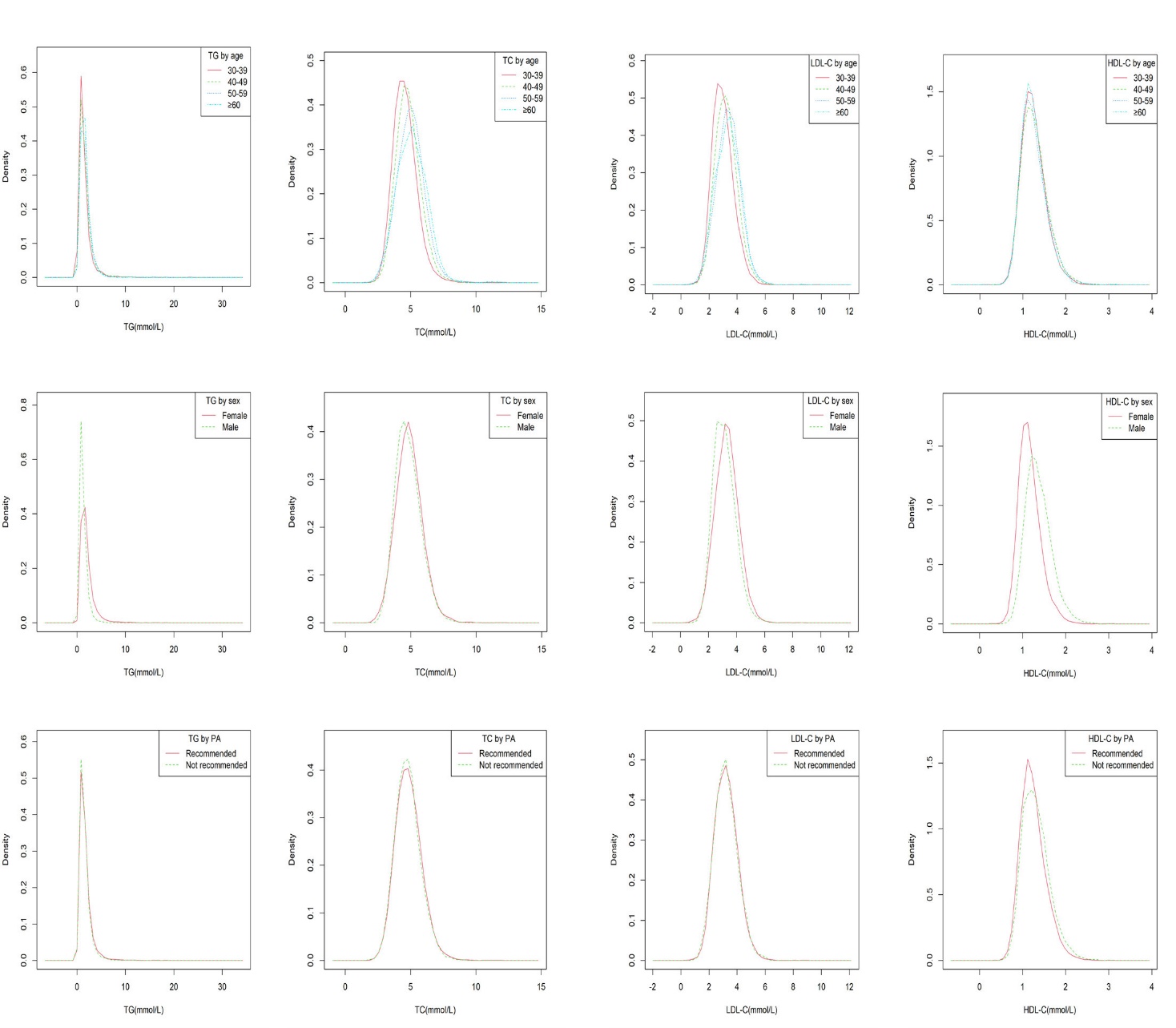


Additional Fig. 1 Kernel density plots for lipid subtypes based on age, sex, and physical activity

**Additional table 3.** Prevalence of different types of dyslipidemia

| **Variables** | **High TC** | **High TG** | **High LDL-C** | **Low HDL-C** | **Dyslipidemia** |
| --- | --- | --- | --- | --- | --- |
| Total, n (%) | 1166 (9.8) | 2324 (19.6) | 1764 (14.9) | 2072 (17.5) | 4661 (39.3) |
| Male | 663 (10.5) | 1820 (28.9) | 1122 (17.8) | 1637 (26.0) | 3313 (52.6) |
| Female | 503 (9.1) | 504 (9.1) | 642 (11.6) | 435 (7.8) | 1348 (24.3) |
| *P* value | <0.001 | <0.001 | <0.001 | <0.001 | <0.001 |
| Age (years) |  |  |  |  |  |
| 30-39, n (%) | 132 (4.5) | 433 (14.7) | 233 (7.9) | 520 (17.7) | 893 (30.4) |
| 40-49, n (%) | 344 (8.2) | 824 (19.6) | 551 (13.1) | 697 (16.6) | 1553 (36.9) |
| 50-59, n (%) | 507 (13.6) | 867 (23.3) | 758 (20.4) | 684 (18.4) | 1741 (46.9) |
| ≥60, n (%) | 183 (18.4) | 200 (20.1) | 222 (22.3) | 171 (17.2) | 474 (47.7) |
| *P* for trend | <0.001 | <0.001 | <0.001 | 0.549 | <0.001 |
| Education, n (%) |  |  |  |  |  |
| college below | 269 (12.4) | 486 (22.4) | 392 (18.1) | 414 (19.1) | 962 (44.3) |
| college and above | 897 (9.3) | 1838 (19.0) | 1372 (14.2) | 1658 (17.1) | 3699 (38.2) |
| *P* value | <0.001 | <0.001 | <0.001 | <0.001 | <0.001 |
| Hypertension, n (%) |  |  |  |  |  |
| Yes | 532 (14.3) | 1100 (29.6) | 759 (20.4) | 825 (22.2) | 1965 (52.9) |
| No | 634 (7.8) | 1224 (15.0) | 1005 (12.4) | 1247 (15.3) | 2696 (33.1) |
| *P* value | <0.001 | <0.001 | <0.001 | <0.001 | <0.001 |
| Diabetes, n (%) |  |  |  |  |  |
| Yes | 165 (17.2) | 350 (36.5) | 216 (22.5) | 281 (29.3) | 590 (61.6) |
| No | 1001 (9.1) | 1974 (18.1) | 1548 (14.2) | 1791 (16.4) | 4071 (37.4) |
| *P* value | <0.001 | <0.001 | <0.001 | <0.001 | <0.001 |
| Overweight/obesity, n (%) |  |  |  |  |  |
| Yes | 812 (11.7) | 1892 (27.3) | 1241 (17.9) | 1674 (24.1) | 3511 (50.6) |
| No | 354 (7.2) | 432 (8.8) | 523 (10.6) | 398 (8.1) | 1150 (23.4) |
| *P* value | <0.001 | <0.001 | <0.001 | <0.001 | <0.001 |
| Smoking |  |  |  |  |  |
| Yes | 319 (11.1) | 955 (33.4) | 537 (18.8) | 837 (29.2) | 1659 (58.0) |
| No | 847 (9.4) | 1369 (15.2) | 1227 (13.6) | 1235 (13.7) | 3002 (33.4) |
| *P* value | <0.001 | <0.001 | <0.001 | <0.001 | <0.001 |
| Physical activity, n (%) |  |  |  |  |  |
| Recommended | 233 (9.5) | 411 (16.8) | 368 (15.0) | 328 (13.4) | 850 (34.7) |
| Not recommended | 933 (9.9) | 1913 (20.3) | 1396 (14.8) | 1744 (18.5) | 3811 (40.5) |
| *P* value | 0.559 | <0.001 | 0.804 | <0.001 | <0.001 |
| Diet quality, n (%) |  |  |  |  |  |
| Poor | 237 (10.5) | 591 (26.2) | 369 (16.3) | 515 (22.8) | 1072 (47.5) |
| Intermediate | 633 (9.8) | 1221 (18.8) | 942 (14.5) | 1140 (17.6) | 2498 (38.6) |
| Good | 296 (9.5) | 512 (16.4) | 453 (14.5) | 417 (13.4) | 1091 (35.0) |
| *P* value | 0.467 | <0.001 | 0.099 | <0.001 | <0.001 |

Data were shown as number (percentages), and Differences between the groups were examined by *X*^2^ test

TC total cholesterol, TG triglyceride, LDL-C low-density lipoprotein cholesterol, HDL-C high-density lipoprotein cholesterol


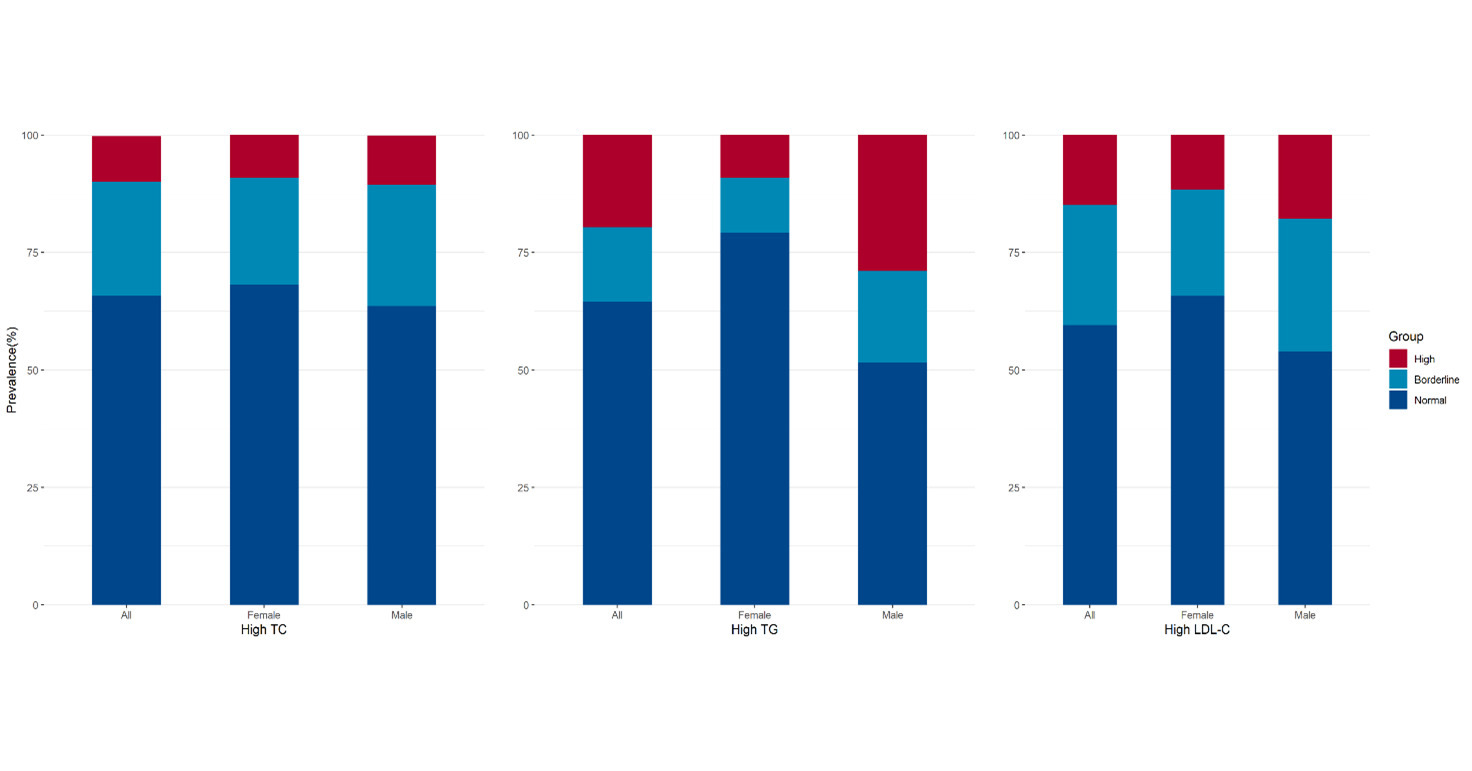


Additional Fig. 2 The prevalence of borderline high and high of lipid subtypes based on sex


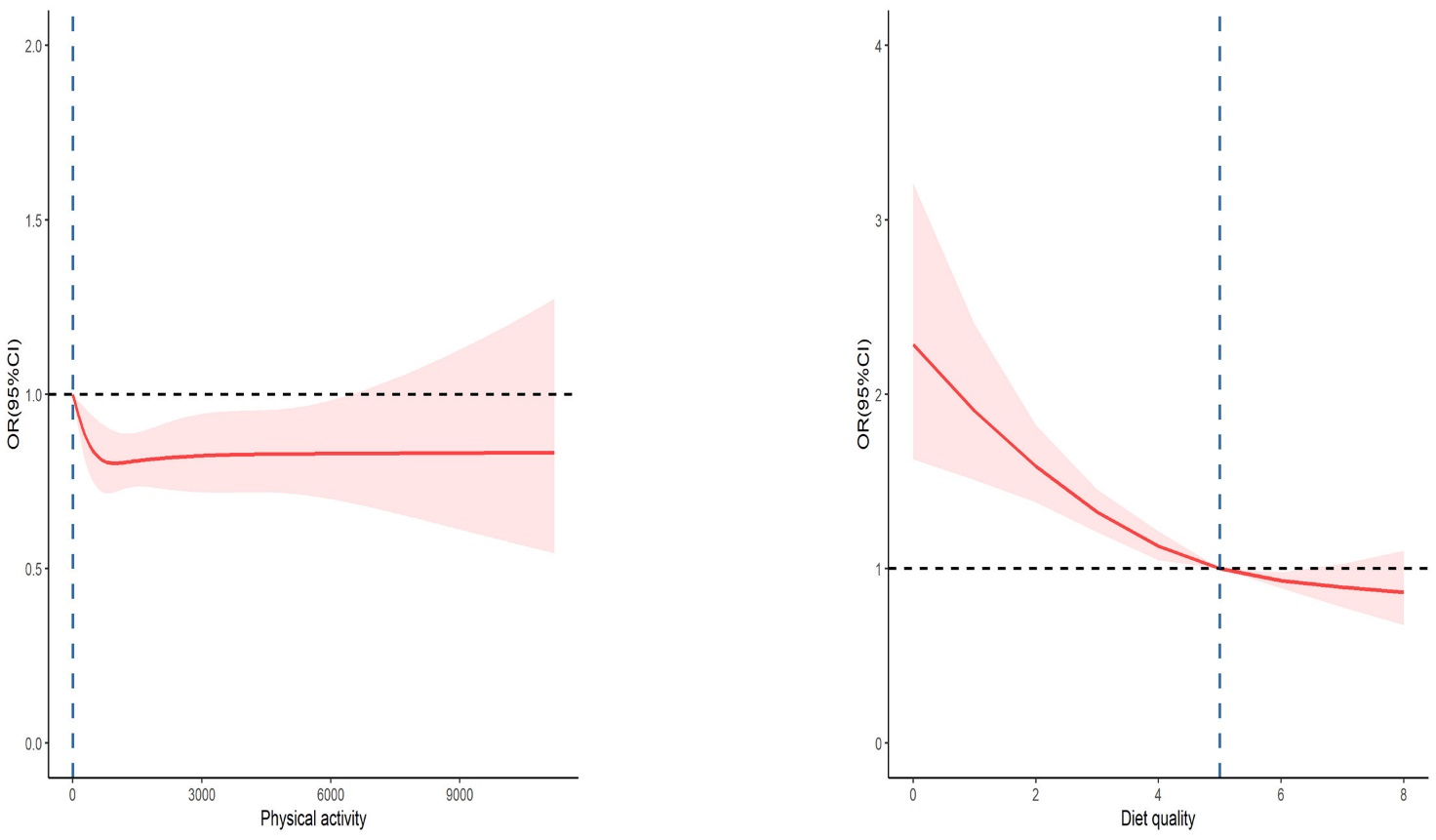


Additional Fig. 3 Dose-response association between moderate-to-vigorous physical activity, diet quality with dyslipidemia

**Additional table 4.** Joint associations of physical activity and diet quality with dyslipidemia

| **Physical activity** | **Diet quality** | **N** | **Case (%)** | **Adjusted OR (95%CI)** | ***P* for interaction** |
| --- | --- | --- | --- | --- | --- |
| Recommended | Good | 790 | 231 (5.0) | 1.00 | <0.001 |
|  | Intermediate | 1321 | 468 (10.0) | 1.229 (1.003-1.505) |  |
|  | Poor | 336 | 151 (3.2) | 1.464 (1.106-1.939) |  |
| Not recommended | Good | 2328 | 860 (18.5) | 1.510 (1.252-1.821) |  |
|  | Intermediate | 5157 | 2030 (43.6) | 1.513 (1.270-1.801) |  |
|  | Poor | 1923 | 921 (19.8) | 1.692 (1.398-2.048) |  |

Multivariable logistic regression adjustment for sex, age, education level, overweight or obesity, diabetes, hypertension, and smoking

OR odds ratio, CI confidence interval

**Additional table 5.** Joint associations of physical activity and age with dyslipidemia

| **Physical activity** | **Age** | **N** | **Case (%)** | **Adjusted OR (95%CI)** | ***P* for interaction** |
| --- | --- | --- | --- | --- | --- |
| Recommended | 30-39y | 559 | 168 (3.6) | 1.00 | <0.001 |
|  | 40-49y | 895 | 292 (6.3) | 1.175 (0.922-1.496) |  |
|  | 50-59y | 808 | 323 (6.9) | 1.536 (1.203-1.962) |  |
|  | ≥60y | 185 | 67 (1.4) | 1.285 (0.811-1.874) |  |
| Not recommended | 30-39y | 2379 | 725 (15.6) | 1.281 (1.035-1.585) |  |
|  | 40-49y | 3313 | 1261 (27.1) | 1.519 (1.236-1.868) |  |
|  | 50-59y | 2907 | 1418 (30.4) | 1.900 (1.541-2.344) |  |
|  | ≥60y | 809 | 407 (8.7) | 2.031 (1.580-2.611) |  |

Multivariable logistic regression adjustment for sex, education level, overweight or obesity, diabetes, hypertension, smoking, and diet quality

OR odds ratio, CI confidence interval

**Additional table 6.** Joint associations of diet quality and age with dyslipidemia

| **Diet quality** | **Age** | **N** | **Case (%)** | **Adjusted OR (95%CI)** | ***P* for interaction** |
| --- | --- | --- | --- | --- | --- |
| Good | 30-39y | 628 | 155 (3.3) | 1.00 | <0.001 |
|  | 40-49y | 1107 | 328 (7.0) | 1.157 (0.913-1.466) |  |
|  | 50-59y | 1060 | 457 (9.8) | 1.792 (1.418-2.264) |  |
|  | ≥60y | 323 | 151 (3.2) | 1.874 (0.811-1.874) |  |
| Intermediate | 30-39y | 1731 | 511 (11.0) | 1.133 (0.908-1.412) |  |
|  | 40-49y | 2291 | 838 (18.0) | 1.384 (1.119-1.712) |  |
|  | 50-59y | 1949 | 907 (19.5) | 1.671 (1.345-2.076) |  |
|  | ≥60y | 507 | 242 (5.2) | 1.730 (1.313-2.279) |  |
| Poor | 30-39y | 579 | 227 (4.9) | 1.410 (1.085-1.833) |  |
|  | 40-49y | 810 | 387 (8.3) | 1.613 (1.264-2.058) |  |
|  | 50-59y | 706 | 377 (8.1) | 1.759 (1.366-2.264) |  |
|  | ≥60y | 164 | 81 (1.7) | 1.572 (1.074-2.301) |  |

Multivariable logistic regression adjustment for sex, education level, overweight or obesity, diabetes, hypertension, smoking， and physical activity

OR odds ratio, CI confidence interval
